# Carotenoid Intake and Serum Concentration in Young Finnish Children and Their Relation with Fruit and Vegetable Consumption

**DOI:** 10.3390/nu10101533

**Published:** 2018-10-17

**Authors:** Marianne Prasad, Hanna-Mari Takkinen, Liisa Uusitalo, Heli Tapanainen, Marja-Leena Ovaskainen, Georg Alfthan, Iris Erlund, Suvi Ahonen, Mari Åkerlund, Jorma Toppari, Jorma Ilonen, Mikael Knip, Riitta Veijola, Suvi M. Virtanen

**Affiliations:** 1 Nutrition Unit, Department of Public Health Solutions, The National Institute for Health and Welfare, PO Box 30, 00271 Helsinki, Finland; hanna-mari.takkinen@uta.fi (H.-M.T.); liisa.uusitalo@helsinki.fi (L.U.); Heli.tapanainen@thl.fi (H.T.); mlovaskainen@gmail.com (M.-L.O.); Suvi.ahonen@uta.fi (S.A.); Mari.Akerlund@uta.fi (M.Å.); Suvi.virtanen@thl.fi (S.M.V.); 2 Faculty of Social Sciences/Health Sciences, University of Tampere, 33014 Tampere, Finland; 3 Genomics and Biomarkers Unit, Department of Health, National Institute for Health and Welfare, 00271 Helsinki, Finland; georg.alfthan@thl.fi (G.A.); Iris.erlund@thl.fi (I.E.); 4 Science Center, Tampere University Hospital, 33521 Tampere, Finland; 5 Department of Physiology, Institute of Biomedicine, University of Turku and Department of Pediatrics, Turku University Hospital, 20520 Turku, Finland; Jorma.toppari@utu.fi; 6 Department of Clinical Microbiology, University of Eastern Finland, 70211 Kuopio, Finland; Jorma.ilonen@utu.fi; 7 Immunogenetics Laboratory, University of Turku, 20014 Turku, Finland; 8 Children’s Hospital, University of Helsinki, Helsinki University Hospital, 00029 Helsinki, Finland; Mikael.knip@hus.fi; 9 Research Program Unit, Diabetes and Obesity, University of Helsinki, Helsinki University Hospital, 00014 Helsinki, Finland; 10 Folkhälsan Research Center, University of Helsinki, 00251 Helsinki, Finland; 11 Department of Pediatrics, University of Oulu, 90014 Oulu, Finland; Riitta.veijola@oulu.fi; 12 Center for Child Health Research, University of Tampere and Tampere University Hospital, 33014 Tampere, Finland

**Keywords:** serum carotenoids, dietary carotenoids, children’s diet, biomarkers

## Abstract

Fruit and vegetable intake has been associated with a reduced risk of many chronic diseases. These foods are the main dietary source of carotenoids. The aim of the present study was to evaluate the associations between dietary intake and serum concentrations of α- and β-carotene in a sample of young Finnish children from the population-based birth cohort of the Type 1 Diabetes Prediction and Prevention (DIPP) Study. The current analysis comprised 3-day food records and serum samples from 207 children aged 1, 2 and 3 years. Spearman and partial correlations, as well as a cross-classification analyses, were used to assess the relationship between dietary intake and the corresponding biomarkers. Serum concentrations of α- and β-carotene were significantly higher among the 1-year-old compared to the 3-year-old children. Dietary intakes of α- and β-carotene correlated significantly with their respective serum concentrations in all age groups, the association being highest at the age of 1 year (α-carotene *r* = 0.48; *p* < 0.001 and β-carotene *r* = 0.47; *p* < 0.001), and lowest at the age of 3 years (α-carotene *r* = 0.44; *p* < 0.001 and β-carotene *r* = 0.30; *p* < 0.001). A cross-classification showed that 72–81% of the participants were correctly classified to the same or adjacent quartile, when comparing the reported dietary intakes and the concentrations of the corresponding carotenoid in serum. The 3-day food record seems to be reasonably valid in the assessment of root vegetable consumption among young Finnish children. Root vegetables were the main dietary source of both carotenoids in all age groups. The high consumption of commercial baby foods among the 1-year-old children was reflected in the relatively high dietary intake and serum concentration of both carotenoids.

## 1. Introduction

Diets rich in fruits and vegetables are associated with reduced risk of obesity [1] and several chronic diseases [2,3,4]. Therefore, many global health promotion programs endorse an increased consumption of plant-based foods [5,6]. Diet-related health problems are often already rooted in childhood, and dietary behaviors adopted early in life tend to continue [7,8], making it particularly important to introduce healthy eating habits from a young age. Patterns of food intake established already during the first year of life tend to persist years later [8]. In Finland, the consumption of vegetables, fruits and berries is remarkably low among young children, and reduces further with age [9,10]. There is a clear need for the promotion of the benefits of fruit and vegetable consumption among young children, and to evaluate diets early.

A number of plant components may be responsible for the protective effects of plant foods [2,11,12]. Carotenoids have been associated with many health benefits, and they offer an ideal biomarker for fruit and vegetable intake; they are available solely through the diet and dietary supplements, and can be reliably analyzed in serum and tissue samples [13,14]. Blood concentrations of carotenoids are known to respond, at least to some extent, to the consumption of carotenoid-containing foods, which includes by and large fruits and vegetables [15]. The major carotenoids that represent over 95% of the total blood carotenoids in human serum include β-carotene, α-carotene, β-cryptoxanthin, lutein, zeaxanthin, and lycopene, of which the first three are provitamin A carotenoids, and they are the most common forms found in the human diet. About half of the provitamin carotenoids in the human intestine are absorbed intact, which the other half can be converted to vitamin A and are mainly stored in the liver as retinyl esters. Several factors from the food matrix and cooking practices to polymorphisms in the genes that code for the proteins that regulate intestinal absorption, result in large individual variability in the metabolism and transport of carotenoids and vitamin A [16,17]. In addition, the existing vitamin A status of the individual has an influence on how efficiently provitamin A carotenoids are converted to retinol [16,17].

To accurately measure the consumption of plant foods, and to evaluate the overall quality of the diet, valid long-term dietary assessment methods are needed. Challenges in assessing young children’s dietary intakes include the day-to-day variation in dietary intakes, changes in dietary habits by age, the fact that the intakes are reported by third persons such as parents or daycare personnel, and part of the food served potentially remaining unconsumed [18,19,20]. Biomarkers, such as serum carotenoids, offer an alternative measurement for the consumption of fruits and vegetables, and they allow the validation of the more traditional dietary assessment methods. However, to date, little research has been carried among children to validate fruit and vegetable intakes, while adult populations have been better described in terms of carotenoid status and plant food intakes. 

Against this background, we determined the main sources of α- and β-carotene in the diet of 1-, 2- and 3-year-old Finnish children. In addition, we assessed the correlations between α- and β-carotene intake from the diet, as reported by 3-day food records, and their respective serum concentrations.

## 2. Materials and Methods

We analyzed data from subjects participating in the Type I Diabetes Prediction and Prevention (DIPP) Nutrition Study, which is part of the larger DIPP Study that started in 1994. The DIPP Study is an ongoing population-based cohort aimed at exploring possible means of predicting and preventing type I diabetes among children with HLA-DQB1 (major histocompatibility complex, class II, DQ beta 1)-conferred susceptibility to the disease. Infants born in the University Hospital areas of Turku, Oulu and Tampere in Finland were enrolled in the DIPP Study by screening cord blood samples for increased genetic susceptibility [21]. The children were monitored for the emergence of type 1 diabetes-associated autoantibodies, growth (body weight and height), diet, and viral infections with clinical visits at the ages of 3, 6, 12, 18, and 24 months, and annually thereafter. Information on parental age and education was obtained through a structured questionnaire that was filled up after delivery. Information on gestational age, number of earlier deliveries and maternal smoking during pregnancy was received from the Medical Birth Registries of the Oulu and Tampere University Hospitals. The Local Ethics Committees approved the protocols, and informed consent was obtained from the parents of the participants.

The subjects for the current study are derived from a subcohort of the DIPP study children born between the 1 October 1996, and 31 August 2004 in the Oulu University Hospital, and between the 20 October 1997 and 5 September 2004 in the Tampere University Hospital (*n* = 5787; 76% of invited children). For the aetiological analyses, cases of pre-type 1 diabetes or clinical type 1 diabetes have been identified from this cohort, and two control children, free of pre- or clinical type 1 diabetes, were randomly selected for each case child, matched for birth date, sex, delivery hospital and genetic risk group [22]. The current analysis comprises these control children (*n* = 207) at the ages of 1, 2 and 3 years. Children who were breast-fed at the age of 1 year (*n* = 47) were excluded from the 1-year age group, because their total dietary intake could not be reliably estimated. A serum sample for carotenoid analyses, which is separate from the sample drawn for the autoantibody follow-up, was collected from all the cohort children at the age of 1 year, and annually thereafter. For the current study, we had a serum carotenoid sample and dietary data available from 135 in the 1-year-olds, 177 in the 2-year-olds, and 141 in the 3-year-olds. 

### 2.1. Dietary Methods

Food intake data were obtained through a 3-day food record, when the child reached the age of 3 and 6 months, and thereafter at the ages of 1, 2, 3, 4 and 6 years. Each year, a food record was collected once from each child, close to the child’s birthday. For the current analysis, food records from the ages of 1, 2, and 3 years were used. The parents, and if the child was taken care by other people, the day-care personnel or other caretakers, recorded the children’s diet, including the use of any dietary supplements, on three consecutive days, of which two were weekdays and one a weekend day. The parents were given face-to-face and written instructions to help them complete the food record, including information on the use of household and weighed measures (e.g., spoons, cups, glasses, and deciliters) and a food picture booklet to help to estimate portion sizes. The day care personnel received written instructions, which included an example of a 1-day food record. Both the parents and the day care personnel were requested to also record the time of the meal, and information on where the food was consumed. At each study center visit, a trained DIPP study nurse or physician checked over the food records and gathered more information where necessary. A trained nutritionist verified the food records and entered them into the food database using Finessi software [23]. The food consumption data collection, and food and nutrient intake calculations have been described in more detail previously [24]. Daily energy and nutrient intakes were calculated using an in-house software linked to the Fineli National Food Composition Database and the Fineli Nutrition Database, both of which are available at the National Institute for Health and Welfare, Finland [23]. Analytical nutrient values of the main carotenoid contributors in the database are mostly based on Finnish studies with additional data obtained from the Finnish food industry and international food composition databases. The DIPP Nutrition Study group has added commercial baby foods and infant formulas to the Fineli Nutrition Database with nutrient content data, including carotenoids [23]. The infant-food database is regularly updated, and it includes all commercial baby foods and infant formulas available in Finland. In the present study, we used the ingredient data, which reports the dietary intakes of ingredient groups such as different types of fruits and vegetables, and also the food level data that allows the analysis of dietary intake as dishes served, such as commercial baby foods.

For the analyses, we selected all ingredient groups consisting of vegetables, fruits, and berries as possible sources of α- and β-carotene. In addition, any ingredient group with a proportional contribution of more than 10% to the intake of either carotenoid in any age group was included. The final ingredient groups in the analyses were: fruits and berries (excluding citrus fruits, fruit juices and juice drinks), citrus fruits, fruit juices and juice drinks, potatoes, root vegetables, green leafy vegetables (including cabbage), other vegetables, and flavored sauces (ketchup and stocks). In addition, we calculated the intake of α- and β-carotene on a food use level, including commercial baby foods as dishes, because of their particularly high rate of consumption among young Finnish children, and the fact that a large proportion of these foods contain fruits and/or vegetables as their main ingredient. The groups (dishes) in at the food use level included commercial baby fruit and berry products (all commercial fruit and berry mashes), commercial baby porridges (milk and/or water-based porridges with/without mashed or powdered fruits and/or berries), commercial baby vegetable products (do not contain fish or meat), commercial baby meat and fish products (all commercial products that contain either meat or fish), and all other dishes as a combined group of “all other dishes” (including all other food use level dishes except for the above-mentioned commercial baby foods). Supplemental intakes were not included in the analyses, as none of the supplements used contained carotenoids.

### 2.2. Determination of Serum Carotenoids and Cholesterol

The methodology used in assessing the serum carotenoid and cholesterol concentrations has been described previously [24]. Briefly, non-fasting venous blood samples were taken by venipuncture for the serum α- and β-carotene, and cholesterol analyses. During processing, the samples were protected from light and kept at room temperature from 30 to 60 min to clot. After centrifugation, a serum sample was taken. Serum samples were stored at −70 °C and transported on dry ice for analyses. Serum α- and β-carotene concentrations were measured using reversed-phase High Performance Liquid Chromatography HPLC at 450 nm [13]. Peak height/internal standard ratios were compared to the ratios of reference plasma, the values of which were traceable to NIST certified serum standards, 986b (National Institute of Standardization and Technology, Gaithersburg, MD, USA). The interassay CV was 9.4%. Serum cholesterol concentration was analyzed manually by an Olli-C photometer (Thermo Fisher Scientific Ltd., Vantaa, Finland) using an enzymatic (CHOD_PAP) method.

### 2.3. Statistical Analyses

Statistical analyses were performed using SPSS software package 18.0 (SPSS Inc., Chicago, IL, USA, 2009). Means and standard deviations (SDs) were tabulated by age group for the dietary intake of α- and β-carotene, total carotenoids, α- and β-carotene serum concentrations, serum cholesterol, and total energy intake. The means of body mass index were calculated for each age group and sex separately (data not shown). The biochemical serum measures of α- and β-carotene and their dietary intakes were skewed, and therefore they were log-transformed prior to analyses. Serum cholesterol was used in the normal scale.

Paired-samples *t*-test was used to examine differences in serum values and dietary intakes by age group. The independent-sample *t*-test was used to examine any differences in serum carotenoid and cholesterol concentrations, and the body mass index, between the sexes (data not shown).

We computed Spearman correlation coefficients to assess the potential associations between α- and β-carotene serum concentrations, and their respective dietary intakes for each age group. We then calculated partial correlation coefficients to describe the associations between serum and diet, adjusted for the effects of energy intake, serum cholesterol, and body mass index that explain some of the variation in the dietary intakes and serum levels of these nutrients. Both α- and β-carotene are mostly transported by low-density lipoproteins in circulation, but as low-density lipoprotein measures were not available for this study, we ran partial correlations with cholesterol to account for some of the serum lipid variation. To assess the difference between the correlation coefficients between age groups, Fischer’s r-to-z transformations were used. 

Furthermore, the ability of the 3-day food record to assign the children correctly by quartiles of α- and β-carotene intake was evaluated using cross-classification analysis using serum concentrations of carotenoids as the reference method. We first calculated the quartile cut-off points for the carotenoids based on both methods separately, and then carried out a cross classification to calculate the percentage of children correctly classified (same or adjacent quartile) or grossly misclassified (categorized into opposite i.e., lowest/highest quartile) by the two methods.

We did not receive serum cholesterol analyses for two children among the 2-year-olds, and one child among the 3-year-olds, and these children were not included in the adjusted analyses. In addition, information on BMI was not available for three children in the 1-year-olds, six children in the 2-year-olds, and seven children in the 3-year-olds, and these children were not included in the adjusted analyses. 

In all analyses, *p* < 0.05 was considered to be significant.

## 3. Results

The present series comprised 129 boys (62.3%) and 78 girls (37.7%), with α- and β-carotene serum samples and food records available from 135 at the age of 1 year, 177 at the age of 2 years, and 141 at the age of 3 years. The background characteristics of the study subjects have been described in more detail elsewhere [24]. Briefly, the distribution of sex, delivery hospital, and genetic risk groups did not differ between the case and control children. The mothers of the case children belonged to the highest age and education categories more frequently than mothers of the control children. Type 1 diabetes in a first-degree relative was more common among the case children than in the controls. The mean α- and β-carotene serum concentrations, as well as their dietary intake and dietary sources, are shown in the untransformed scale (Table 1 and Table 2).

Serum levels of both α- and β-carotene were significantly higher at the age of 1 year compared to the ages of 2 and 3 years. There was a significant decrease in the age-specific mean concentration of α-carotene from 0.50 mmol/L in the 1-year-olds to 0.25 mmol/L in the 3-year-olds (*p* < 0.001), and of β-carotene from 1.25 mmol/L in the 1-year-olds to 0.74 mmol/L in the 3-year-olds (*p* < 0.001). There was also a significant increase in serum cholesterol concentration from 1 to 3 years (*p* < 0.001). The estimated average dietary intake of α-carotene was significantly higher at the age of 1 year compared to 2 and 3 years, while the intake of dietary β-carotene was significantly lower among the 2-year-olds compared to the other age groups. The intake of energy differed significantly between the age groups, increasing with age. Apart from serum cholesterol, which at the age of 2 years was significantly higher among the boys than the girls (*p* = 0.016), there were no differences in the mean concentrations of the other measures by sex (data not shown).

There were no significant differences between the age groups in the consumption of fruits and berries and root vegetables, but the consumption of citrus fruits (*p* < 0.001) and fruit juices and juice drinks (*p* < 0.001) increased significantly from 1 to 3 years. The consumption of potatoes decreased significantly from 1 to 3 years (*p* < 0.01), as did the intake of green leafy vegetables (*p* < 0.01), while the intake of other vegetables increased from the age of 1 year to the age of 3 years (*p* < 0.001). The consumption of flavored sauces was significantly higher among the 2- and 3-year-olds compared to the 1-year-olds.

The main ingredient contributing to the intake of α- and β-carotene in all age groups was root vegetables (Table 3). Flavored sauces, including ketchup and other vegetable concentrates, contributed increasingly to the intake of β-carotene from 0.08% in the 1-year-olds to 11.4% in the 3-year-olds. The proportional contribution of commercial baby foods to the intake of both carotenoids was at its highest at the age of 1 year. In the 1-year-olds, commercial baby fruit and berry purees, baby porridges, baby meat and fish mashes, and baby vegetable products, contributed 51.7% to the total dietary intake of α-carotene, and 51.1% to the total dietary intake of β-carotene. Although the consumption of commercial baby foods decreased considerably with age, commercial baby foods still contributed 9.1% to the total dietary intake of α-carotene and 6.6% to the total dietary intake of β-carotene at the age of 3 years.

The total dietary intake of both carotenoids correlated significantly with the corresponding serum concentration in all the age groups (Table 4). The crude correlation between the intake and serum concentration of α-carotene varied from 0.50 (*p* < 0.01) in the 1-year-olds, to 0.45 (*p* < 0.01) in the 2- and 3-year-olds, and that of β-carotene from 0.47 (*p* < 0.01) in the 1-year-olds to 0.33 (*p* < 0.01) in the 3-year-olds. Although the correlations remained similar after adjustment for factors known to affect serum concentrations of these carotenoids, the p-values improved strengthening the observed correlations. When we applied Fisher’s r-to-z transformation to the resultant correlation coefficients, we observed a significant difference (*p* < 0.05) in the adjusted correlation in the β-carotene diet-serum relationships between the 1- and 3-year-olds. No other significant differences in the correlations between the age groups were observed (data now shown).

When examining cross-classification results, the percentage of children classified into the same or adjacent groups ranged between 78% and 81% for α-carotene, and between 72% and 79% for β-carotene (Table 4). Children who were grossly misclassified i.e., ranked in the first quartile with a 3-day food record and in the fourth quartile with serum concentrations, ranged from 4% to 13%. 

## 4. Discussion

Carotenoids furnish plants with a wonderful range of colors, and as a surplus, possible health benefits. The consumption of carotenoid-containing foods is assumed to be reflected in the serum concentration of carotenoids, at least to some extent. However, the ability to capture a person’s carotenoid status using one dietary assessment method is limited. Both the dietary intake of carotenoids and the serum levels of carotenoids show high within- and between-person variability [25,26]. Our aim was firstly to determine the main dietary sources of α- and β-carotene among young Finnish children, then to examine the associations between the dietary intakes of α- and β-carotene with their corresponding biomarkers, and finally, to conclude whether these two carotenoids could be reliable biomarkers of plant food intake among young children. Our study demonstrated moderate, significant correlations between the dietary intake of both carotenoids and their respective serum concentration in all of the age groups studied. The associations we observed are similar to or stronger than those published previously [2,19,25,26,27,28,29,30,31,32,33,34,35,36,37,38,39]. Among the ingredients, root vegetables were the strongest contributors to the serum levels of both carotenoids in all age groups. Both parents and alternate caregivers, such as day care personnel, provided data for the food records. It has previously been suggested that including alternate caregivers allows a more complete picture of a child’s diet than just parents alone [18]. Our results further show the accuracy of the α- and β-carotene information in the food database used. 

The primary strength of the present study was the use of serum values and food records that were prospective longitudinal measurements rather than cross-sectional measurements allowing us to describe the relationship between age and serum carotenoid levels. A further strength was the use of two different methods to evaluate fruit and vegetable intake; both the 3-day food records, which reflect shot-term intake, as well as the serum concentrations of carotenoids, which reflect both short- and long-term intake. Serum nutrients can undergo two different phases in nutrient transportation, i.e. being transferred from the gut lumen to tissue pools, and later on, being carried away from these tissue pools to be utilized by their final target tissues. This first process reflects more short-term fluctuations in dietary intake, and the latter one, long-term fluctuations [27,40]. Finally, we benefited from a food database with accurate information on the commercial baby foods on the market, and a system that accommodates for the creation of new recipes and the modification of recipes in the food database. 

As for the limitations of this study, we would have profited from having a larger variety of carotenoids studied to assess the best carotene biomarkers of vegetable and fruit intakes overall, and to assess which carotenoids are derived from which ingredient groups. Although the children in the study carry increased HLA-conferred susceptibility to type 1 diabetes, they are expected to represent the general population of young Finnish children. Almost 20% of the Finnish population carries increased HLA-conferred predisposition to type 1 diabetes, while only 3–4% of these individuals progress to the clinical disease [41]. The parents received information about their child’s increased susceptibility to type 1 diabetes when their child was born, which may have resulted in some changes in the family’s lifestyle, such as alternations to childhood diet [42]. Since the serum samples were not fasting samples, and they were collected throughout the year, the seasonality of intake has an influence on serum levels, and it introduces bias in ours study. However, as we had a very similar presentation of children from each season, the influence of season to our results should be rather low.

Previous studies have reported a wide variation and a significant correlation between dietary intakes of α- and β-carotene, and their corresponding biomarkers in adults and adolescents, and a few correlations in children have also been reported [19,20,25,26,27,28,29,30,31,32,33,34,35,36,37,38,39].The correlation coefficients that we observed were close to the high end of the range for both carotenoids, and they compare well with the previous data available on children, and as in earlier studies, we also observed stronger correlations for α-carotene than for β-carotene. In a study among U.S children aged 1 to 3 years, significant correlations were reported between the intake and serum concentrations for both β-carotene (*r* = 0.13) and α-carotene (*r* = 0.39) [30]. The correlation coefficient values were not age-specific, and hence, a direct comparison to the current results is difficult. A Danish study among children aged 8 to 11 years reported significant correlations for both β-carotene (*r* = 0.33) and α-carotene (*r* = 0.54) [29], similar to our results, as did a Norwegian study among children aged 8 to 14 years, with coefficients ranging from *r* = 0.26 to 0.45 for α-carotene and *r* = 0.34 to 0.48 for β-carotene [39]. Body weight has been suggested as a confounder, with higher weights associated with lower plasma carotenoid concentrations [19,35]. The importance of adjusting for the body mass index was seen in a sample of largely overweight US children aged 5 to 12 years. Correlations for β-carotene (*r* = 0.17 crude and *r* = 0.56 adjusted for BMI) and for α-carotene (*r* = 0.25 crude and *r* = 0.51 adjusted for BMI) were strongest after adjustment [19]. Adjusting for body mass index in the current analyses increased the statistical significance, but not the correlation coefficients.

The interpretation of biomarkers as a measure of their dietary intake is influenced by a large range of exogenous and endogenous determinants. Some of the variability in results between individual studies can also be explained by the diversity of plant-based foods consumed in different geographic locations, which results into regional variations in serum carotenoid profiles, and in differences in the ideal serum carotenoid markers of plant food consumption. Among the younger populations, lycopene and β-carotene have been found to be the most abundant plasma carotenoids in the US and Costa Rica [34,38], and lutein in Japan [33]. The high consumption of carrots among Swedish adults is reflected in higher levels of plasma α-carotene, while the concentrations of β-cryptoxanthin, lycopene, and zeaxanthin dominate in Spanish adults [37]. Similarly, the correlations between dietary intakes and plasma levels for α-carotene were higher in Sweden, and those for β-cryptoxanthin were higher in Spain. Carrots are a rich source of α- and β-carotene, and a major component of many of the commercial baby foods available in Finland, and it is also used commonly as the main vegetable ingredient in home-prepared baby meals. This may explain the high correlations that we observed between the diet and serum levels of carotenoids in the 1-year-olds. In general, α- and β-carotenes tend to inter-correlate, as both carotenoids are found in many of the same foods [28].

Variability in absorption and metabolism, co-consumption of inhibitors and promoters, and the processing of the food source, all have substantial influences on carotenoid bioavailability and serum concentrations, and hence they bring further variability in correlation studies. In the case of some carotenoids, mechanical homogenization and heat treatment, which takes place in the preparation of most baby foods, can enhance carotene bioavailability from vegetables considerably [43,44,45,46,47]. This is especially relevant for β-carotene, as the plant matrix in which it is located in vegetables, is considered to be a major limiting factor for its bioavailability [44,45,46,47]. The intensity and duration of the heating process of the carotenoid food source appears to be directly related to β-carotene bioavailability [43,44,45,46,47]. In a study by Rock et al. [47] plasma β-carotene response to raw vs. processed carrots and spinach resulted in a significantly greater amount of both total and all-*trans*- (but not *cis*-, which has been reported to be less bioavailable) plasma β-carotene concentrations after a processed food feeding period, compared to feeding the same foods raw. Plasma concentration of α-carotene, on the other hand, increased significantly in response to both raw and processed vegetable feeding regimens [47]. We observed no significant differences in the dietary intake of root vegetables between the age groups, or that of β-carotene intake from the diet between the 1- and 3-year-olds. However, the correlation coefficients for serum β-carotene with its dietary intake declined from 1 to 3 years of age, albeit with borderline statistical significance. We do suspect, however, that this may reflect the transition from commercial baby foods and other cooked and mashed plant foods to family meals, which may contain for example, less cooked carrots and more raw carrots as such, or in mixed salads (grated raw carrots are an especially common part of mixed salads in Finland). These changes may not be as apparent for α-carotene, which is often more easily absorbed from plant matrix. An earlier study by Erkkola et al. [48] that comprised children from the same cohort as the current study, found that the ratio of within-subject variances to between-subject variances among young children increases with age for a large number of macro- and micronutrients, The study indicated that the ratio of within-subject variances to between-subject variances among preschoolers increases with age. In the present study, we received a stronger correlation between dietary carotenoid intake and serum level among the 1-year-olds, which may reflect also the fact that the 3-day food record is sufficient to assess the intake of α- and β-carotene with higher accuracy in the 1-year-olds than in the older age groups, in which more recording days may be required to achieve the same degree of confidence. 

The average proportion of children classified in the same or adjacent group in our study ranged between 72% and 81%, while between 4% and 13% were grossly misclassified into the opposite group. This demonstrated a moderate agreement between the two methods of assessment, and agrees with earlier studies carried out with carotenoids among older children [39]. It is known that cross-classification analyses can group subjects with very different intakes into one category, and also subjects with similar intakes into a different categories if they are close to the cut-off point, especially if the number of subjects in the study is small. In a small study, like ours, a few subjects can make a large difference to the percentages. We considered the classification of children in corresponding quartiles of carotenoid intakes reported in the 3-day food records and serum levels as being reasonably accurate (approximately 70%). 

All current methods for assessing dietary intakes have their limitations. Blood carotenoids as a biomarker can overcome the errors of self-reported intake and bias introduced by the use of food composition tables. However, biomarkers themselves also have limitations, due to many inter-individual factors that contribute to variation in biomarker levels, and hence biomarkers do not only reflect differences in dietary intake. In the current study, we used the circulating concentration of α- and β-carotene as biomarkers to validate the reported intake of α- and β-carotene from food. There are also other novel technological methods to assess the intake of fruits and vegetables through carotenoid intakes that completely avoid the collection of biological specimens. One such non-invasive method is the use of Raman spectroscopy, which measures skin carotenoid status. Studies have already been conducted among adults, and resulted in strong correlations with total plasma carotenoids. Evidence among children is also promising [49]. 

## 5. Conclusions

In summary, according to data from 3-day food records, α- and β-carotene appear to be reasonably valid biomarkers of corresponding carotene intakes among young children in Finland. The high consumption of commercial baby foods in Finland is reflected in the high serum levels of α- and β-carotene among the 1-year-olds. Our results indicate that root vegetable intake is a significant determinant of serum α- and β-carotene levels among Finnish children in early childhood, and that a high consumption of commercial baby foods is reflected in the higher serum carotenoid levels in the young age groups. Our results show the high quality of the 3-day food records kept by the parents and the day care personnel, and the accuracy of the α- and β-carotene information in the food databases used.

## Figures and Tables

**Table 1:** Serum α-and β-carotene and cholesterol concentration in 1-, 2-, and 3-year-old Finnish children.

	1-year-olds (*n* = 135)		2-year-olds ^a^ (*n* = 177)		3-year-olds ^b^ (*n* = 141)	
	Mean (SD)	*p*^c^ (Age Groups 1 vs. 2)	Mean (SD)	*p*^c^ (Age Groups 2 vs. 3)	Mean (SD)	*p*^c^ (Age Groups 1 vs. 3)
Biomarkers						
Serum cholesterol (mmol/L)	4.3 (0.76)	<0.01	4.6 (0.82)	NS	4.7 (0.83)	<0.001
Serum α-carotene (µmol/L)	0.50 (0.38)	<0.001	0.25 (0.29)	NS	0.25 (0.25)	<0.001
Serum β-carotene (µmol/L)	1.25 (1.02)	<0.001	0.76 (0.70)	NS	0.74 (0.56)	<0.001
Dietary intake						
α-carotene (µg/day)	672 (743)	<0.001	451 (550)	NS	505 (559)	<0.001
β-carotene (µg/day)	1579 (1497)	<0.01	1280 (1210)	<0.05	1515 (1337)	NS
Total carotenoids ^d^ (μg/day)	3245 (2611)	NS	3262 (2532)	<0.05	3920 (2602)	NS
Intake of energy (kJ/day)	3764 (660)	<0.001	4618 (881)	<0.001	5153 (959)	<0.001

^NS^ Nonsignificant *p*-values, *p* > 0.05. ^a^ Serum cholesterol sample missing from two children (*n* = 175). ^b^ Serum cholesterol sample missing from one child (*n* = 140). ^c^ Differences between the means by age group were tested with the dependent samples *t*-test. ^d^ Total carotenoids include α-carotene, β-carotene, cryptoxanthin, γ-carotene, canthaxanthin, capsaicin, lutein, zeaxanthin, and lycopene.

**Table 2:** Estimated daily dietary intake of α- and β-carotene food sources as ingredients in 1-, 2-, and 3-year-old Finnish children.

Food Intake as Ingredients (g/day)	1-year-olds (*n* = 135)		2-year-olds (*n* = 177)		3-year-olds (*n* = 141)	
	Mean (SD)	*p*^a^ Age Groups 1 vs. 2	Mean (SD)	*p*^a^ Age Groups 2 vs. 3	Mean (SD)	*p*^a^ Age Groups 1 vs. 3
Fruits and berries (other than citrus)	63.6 (42.6)	NS	65.5 (50.8)	NS	63.0 (56.5)	NS
Citrus fruits	1.1 (7.2)	<0.001	7.9 (18.8)	NS	9.6 (21.3)	<0.001
Fruit juices and juice drinks	28.4 (68.4)	<0.001	152.1 (142.4)	<0.001	192.4 (147.4)	<0.001
Potatoes	73.8 (42.7)	<0.05	61.2 (42.5)	NS	55.2 (39.5)	<0.01
Root vegetables	19.7 (22.6)	NS	16.7 (19.7)	NS	18.0 (19.3)	NS
Green leafy vegetables	4.2 (10.7)	<0.01	2.5 (5.6)	NS	2.7 (7.5)	<0.01
Other vegetables	17.1 (15.9)	<0.001	28.1 (27.9)	NS	33.1 (33.8)	<0.001
Flavored sauces ^b^	3.3 (12.9)	<0.001	9.2 (16.8)	NS	9.7 (15.7)	<0.01

^NS^ Nonsignificant *p*-values, *p* > 0.05. ^a^ Differences between the means by age group were tested with the dependent-samples *t*-test. ^b^ Flavored sauces include mustard, ketchup, soya sauce, and beef, chicken, fish, and vegetable stocks.

**Table 3:** Proportional contribution (%) of dietary sources to the intake of α- and β-carotene by age group.

Carotenoid Intake Contributors	α-Carotene			β-Carotene		
	1-Year-Olds (*n* = 135)	2-Year-Olds (*n* = 177)	3-Year-Olds (*n* = 141)	1-Year-Olds (*n* = 135)	2-Year-Olds (*n* = 177)	3-Year-Olds (*n* = 141)
Food intake as ingredients (g/day)						
Fruits and berries (other than citrus)	0.61	0.90	0.80	3.4	2.6	1.5
Citrus fruits	0.03	0.30	0.32	0.02	0.20	0.20
Fruit juices and juice drinks	0.05	0.67	2.2	0.15	1.1	1.8
Potatoes	0.00	0.00	0.00	0.33	0.35	0.27
Root vegetables	82.1	87.2	89.9	73.8	67.1	65.7
Green leafy vegetables	0.28	0.24	0.23	0.70	1.8	2.6
Other vegetables	1.4	3.0	1.9	4.7	7.1	6.4
Flavored sauces ^a^	0.01	0.54	0.98	0.08	6.5	11.4
Miscellaneous ^b^	17.5	7.2	3.7	16.9	13.2	10.2
All ingredients	100	100	100	100	100	100
Food intake as dishes (g/day)						
Commercial baby fruit and berry purees	0.62	0.06	0.09	2.7	0.53	0.20
Commercial baby porridges ^c^	15.4	0.65	0.34	13.5	0.44	0.24
Commercial baby vegetable mashes	4.0	3.9	3.3	4.5	3.2	2.3
Commercial baby meat and fish mashes ^d^	31.7	11.2	5.4	30.4	9.3	3.9
All other dishes	48.3	84.2	90.9	48.9	86.5	93.4
All dishes	100	100	100	100	100	100

^a^ Flavored sauces include mustard, ketchup, soya sauce, and beef, chicken, fish, and vegetable stocks. ^b^ Miscellaneous foods in the 1-year-olds consist mostly of homemade baby foods. ^c^ Commercial baby porridges also contain porridges with added fruits and berries either as mashes or powders. ^d^ Commercial baby meat and fish products have a high proportion of root vegetables as ingredients, most often carrots.

**Table 4:** Spearman and partial correlation coefficients (adjusted for energy intake, serum cholesterol and body mass index), and cross-classification averages of quartiles, estimated from the daily dietary intakes of α-and β-carotene, assessed with 3-day food records and the respective serum concentrations among 1-, 2-, and 3-year-old Finnish children.

Dietary Intake, µg/day	Crude	Adjusted ^d^	Quartiles, % Within One Quartile	Opposite Quartile
1-year-olds ^a^ (*n* = 135)				
Total dietary α-carotene ^d^	0.50 **	0.48 ***	81	7
Total dietary β-carotene ^d^	0.47 **	0.47 ***	79	6
2-year-olds ^b^ (*n* = 177)				
Total dietary α-carotene ^d^	0.45 **	0.45 ***	79	9
Total dietary β-carotene ^d^	0.34 **	0.40 ***	74	11
3-year-olds ^c^ (*n* = 141)				
Total dietary α-carotene ^d^	0.45 **	0.44 ***	78	4
Total dietary β-carotene ^d^	0.33 **	0.30 ***	72	13

*p*-value < * 0.05, < ** 0.01, < *** 0.001. ^b^ Serum cholesterol sample missing from two children (*n* = 175) and body mass index measurement from six children (*n* = 169). ^c^ Serum cholesterol sample missing from one child (*n* = 140), and body mass index measurement from seven children (*n* = 133). ^d^ Partial correlation coefficients adjusted for serum cholesterol concentration, energy intake, and body mass index.
